# Numerical analysis of 2.7 μm lasing in Er^3+^-doped tellurite fiber lasers

**DOI:** 10.1038/srep31761

**Published:** 2016-08-22

**Authors:** Weichao Wang, Lixiu Li, Dongdan Chen, Qinyuan Zhang

**Affiliations:** 1 State Key Lab of Luminescent Materials and Devices and Institute of Optical Communication Materials, South China University of Technology, Guangzhou 510640, P. R. China; 2 Guangdong Engineering Technology Research and Development Center of Special Optical Fiber Materials and Devices, South China University of Technology, Guangzhou 510640, P. R. China; 3 Guangdong Provincial Key Lab of Fiber Laser Materials and Applied Techniques, South China University of Technology, Guangzhou 510640, P. R. China

## Abstract

The laser performance of Er^3+^-doped tellurite fiber lasers operating at 2.7 μm due to ^4^I_11/2_ → ^4^I_13/2_ transition has been theoretically studied by using rate equations and propagation equations. The effects of pumping configuration and fiber length on the output power, slope efficiency, threshold, and intracavity pump and laser power distributions have been systematically investigated to optimize the performance of fiber lasers. When the pump power is 20 W, the maximum slope efficiency (27.62%), maximum output power (5.219 W), and minimum threshold (278.90 mW) are predicted with different fiber lengths (0.05–5 m) under three pumping configurations. It is also found that reasonable output power is expected for fiber loss below 2 dB/ m. The numerical modeling on the two- and three-dimensional laser field distributions are further analyzed to reveal the characteristics of this multimode step-index tellurite fiber. Preliminary simulation results show that this Er^3+^-doped tellurite fiber is an excellent alternative to conventional fluoride fiber for developing efficient 2.7 μm fiber lasers.

Rare-earth-doped fiber lasers operating at mid-infrared (MIR) regions have attracted plenty of attention due to a wide range of applications, including eye-safe radars, medical surgery, remote sensing, and military weapons^1^,^2^,^3^. The ^4^I_11/2_ → ^4^I_13/2_ radiative transition allows Er^3+^ to be a promising candidate activator for such a laser operation. However, the narrow energy gap between these two levels makes this emission very susceptible to the phonon energy of the host, thus the current reported glassy matrix for Er^3+^ laser at 2.7 μm is only limited to fluoride glass fibers (e.g. ZBLAN glass). Although a record output power of 30.5 W has been achieved from an Er^3+^-doped fluoride fiber laser^4^, the weaker bond strength and lower optical damage threshold of ZBLAN glass severely restricts the attainable peak power levels. In an effort to find viable alternatives, researchers turn their attention from fluoride glass to multi-component oxide glass with lower phonon energy and moderate strength. Until now, the 2.7 μm fluorescence of Er^3+^ has been frequently reported from many oxide glass host (e.g. silicate, fluorophosphate, germanate, and tellurite glasses), but there has been no report on their fiberization and laser operation^5^,^6^,^7^,^8^. Therefore, it is extremely essential to develop a multi-component oxide glass with superior physical, mechanical, and photoluminescence performance from the perspective of practical application.

As a promising alternative, tellurite glass plays an important part in fiber lasers, amplifiers, waveguide, and photonic crystal fiber for its unique optical and physical properties, such as lower phonon energy, better thermal ability, etc.^9^,^10^. Tellurite-based lasers operating from 1.0 to 2.1 μm with various shapes such as bulk glasses, microspheres, and fibers have been realized in the past decades, which demonstrated its great development potentiality and flexibility in the field of optoelectronic functional materials^11^,^12^,^13^,^14^. Recently, we achieved a broadband 2.7 μm amplified spontaneous emission from the Er^3+^-doped tellurite fibers (EDTF)^15^, the desirable spectroscopic characteristics along with excellent thermal stability and mechanical properties indicate that 2.7 μm laser is highly expected in this kind of tellurite glass fiber. To further explore the 2.7 μm laser performance from this Er^3+^-doped tellurite glass fiber, it is essential to carry out a theoretical simulation through the establishment of numerical modeling.

In this paper, a theoretical model for Er^3+^-doped tellurite fiber lasers is established based on the rate equations and propagation equations. The effects of pump configuration and fiber length on the laser output power, slope efficiency, threshold, and intracavity pump and laser power distributions have been systematically investigated to optimize the performance of fiber lasers. The laser performance of conventional Er^3+^-doped ZBLAN and the present tellurite fiber lasers is also compared and analyzed in detail. Furthermore, the two- and three-dimensional laser field distributions are further carried out to reveal the characteristics of this multimode step-index tellurite fiber. This work might be helpful to design tomorrow’s high-efficient 2.7 μm multi-component oxide fiber lasers.

## Numerical Simulation

**Figure 1:**
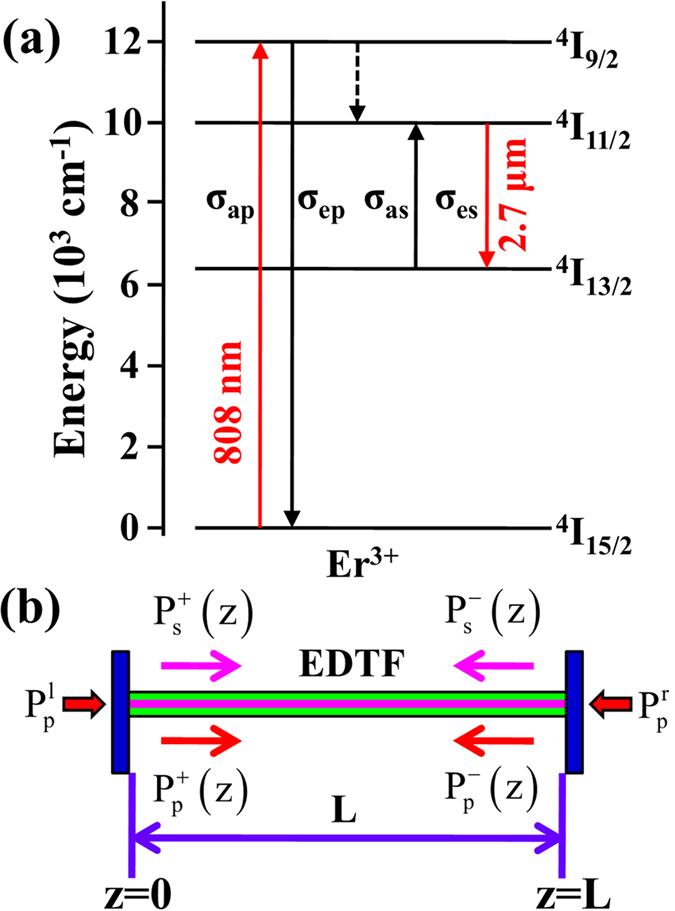
(a) Simplified energy level diagram of Er^3+^. (**b**) End-pumped fiber laser schematic diagram.

Generally, it is extremely difficult to assess the potential of a fiber laser by means of laser tests alone. This is especially true when more than one activator species are incorporated into a laser material. To obviate this difficulty, sophisticated and detailed spectroscopic measurements are performed on the laser host material. Parameters such as absorption and emission cross sections, doping concentration, and lifetimes are essential to solve the complex equations. Using these basic parameters in a complicated laser model, the performance of fiber lasers can be predicted in advance. Laser model has been developed at elsewhere for this purpose and predicted performance can be further compared with experiments to produce a comprehensive understanding of the laser^16^. Previously, Mescia *et al*. designed a simulation model based on a tapered fiber and an Er^3+^-doped chalcogenide microsphere, which can be used to develop high efficiency and compact mid-infrared amplifiers^17^. Similarly, a theoretical model for Er^3+^-doped tellurite fiber lasers can also be established according to the rate equations and propagation equations. Figure 1 shows the simplified energy level diagram of Er^3+^ and end-pumped fiber laser schematic diagram. The model mainly considers four manifolds in Er^3+^, including ^4^I_15/2_, ^4^I_13/2,_^4^I_11/2_, and ^4^I_9/2_ levels. Upon excitation of 808 nm LD, the electrons in Er^3+^: ^4^I_15/2_ ground state is excited to the ^4^I_9/2_ level. After that, non-radiative relaxation from ^4^I_9/2_ state to the ^4^I_11/2_ level occurs and the electrons in ^4^I_11/2_ state partly radiatively relax to ^4^I_13/2_ with emitting a 2.7 μm (λ_s_) emission^18^. The relevant transitions among these levels are shown in Fig. 1(a). In this case, the rate equations and propagation equations for the four levels can be written as^19^.


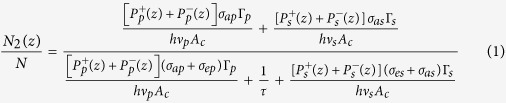



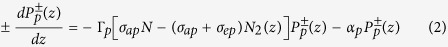



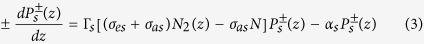
where 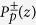 and 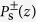 are the pump and signal light powers which propagate along the positive (superscript “+”) or negative (superscript “−”) z direction (0 ≤ z ≤ L, L is the fiber length), respectively. Γ_p_ and Γ_s_ are the filling factor of pump and signal light, respectively. σ_ap(s)_ and σ_ep(s)_ are the absorption and emission cross sections of pump (signal) light, respectively. N_2_ is the population density in ^4^I_11/2_ level and N is the Er^3+^-doping concentration. α_p_ and α_s_ represent the scattering loss of signal and pump light, respectively. h is the Planck constant and A_c_ is the cross-section area of the fiber core. ν_s_ and ν_p_ are the pump and signal frequency, respectively. τ is the lifetime of upper lasing level. The pump and signal photon fluxes are subject to the following boundary conditions at the fiber ends^19^:


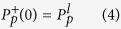



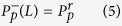



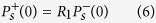



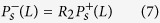
where 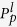 and 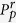 are the pump powers coupled into the fiber from the left and right directions, respectively. R_1_ and R_2_ are the power reflectivities of the front and back cavity mirrors, respectively. These basic physical parameters listed in Table 1 are used to solve the above equations by fourth-order Runge-Kutta algorithm. In the modeling, we assume the fiber lengths are ranging from 0.05 to 5 m, the maximum pump power is 20 W, and the power reflectivities of front and back cavity mirrors remain unchanged in different pumping configurations.

## Simulation Results and Discussion

### Forward pumping configuration

**Figure 2:**
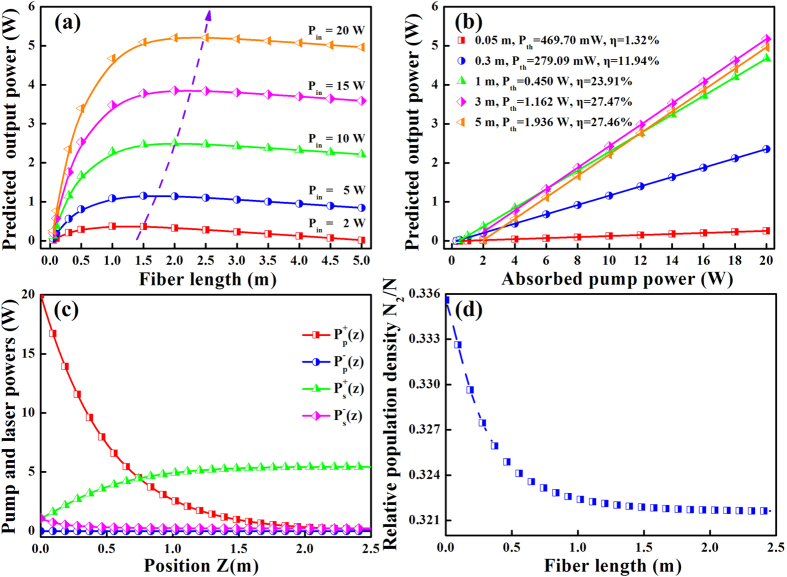
Output power versus (**a**) fiber length for different pump powers, and (**b**) pump power for different fiber lengths. (**c**) The distribution of pump and laser powers, as well as (**d**) the relative population density along the fiber length in forward pumping configuration.

Figure 2(a) depicts the effect of fiber length on output power for different launched pump powers in a forward pumping configuration. It can be found that the output power enhances significantly with increasing fiber length when L < 1 m, which indicates that sufficient amounts of pump powers are absorbed by the fiber with short length. With further increase of fiber length, the output power slightly decreases due to the significant background loss in the fiber. Therefore, the output characteristics of the 1.5 to 2.5 m case are optimal when the input pump power is below 20 W. It should be mentioned that the optimal value of fiber length increases with the increasement of pump power, thus it can speculate that the optimal fiber length will move to larger values if the pump power further increases. The output power as a function of launched pump power with different fiber lengths is plotted in Fig. 2(b). The output power increases with continuously increasing launched pump power, which presents a good linear relationship. When increasing the fiber length from 0.05 to 5 m, the slope efficiency and maximum laser output power both improve enormously by about 20 times, from 1.32% and 0.259 W to 27.47% and 5.209 W, respectively, while the threshold pump power only increases by about 7 times, from 279.09 mW to 1.936 W. Meanwhile, it is noted that the slope efficiency enhances rapidly in the beginning, finally it rises slowly and even keeps constant. The distribution of pump and laser powers along the 2.5 m long fiber is analyzed when the pump power is 20 W, as shown in Fig. 2(c). The forward propagating laser power reaches a maximum near the end of the fiber, where the forward pump power is too low to further amplify the signal. The gradually attenuation of pump power is ascribed to the enhanced influence of scattering and absorption loss. In contrast, the backward signal and pump laser are negligible in the entire fiber. Figure 2(d) displays the relative population density along the optical fiber. As can be seen, the population density decreases monotonically along the fiber length, namely the gain will saturate and cannot increase any more when the fiber length reaches a certain value.

### Backward pumping configuration

**Figure 3:**
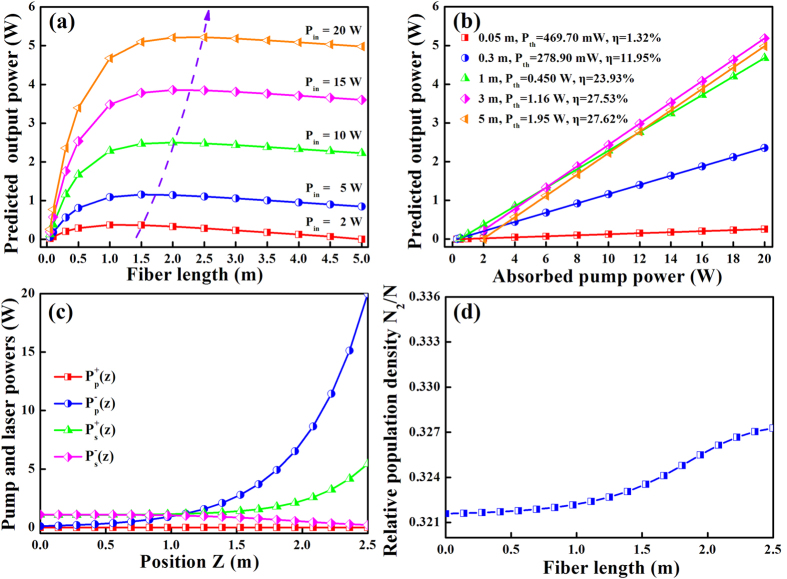
Output power versus (**a**) fiber length for different pump powers, and (**b**) pump power for different fiber lengths. (**c**) The distribution of pump and laser powers, as well as (**d**) the relative population density along the fiber length in backward pumping configuration.

Figure 3 draws the connections among laser output power, fiber length, pump power, the distributions of pump and laser powers, and relative population density along the optical fiber in a backward pumping configuration. The laser output power and the optimal fiber length show a similar variation tendency to the forward pumping configuration. However, the maximum laser output power and slope efficiency of the backward pumping configuration is somewhat greater than that of the forward one. Moreover, the distribution of pump and laser powers and relative population density along the optical fiber also exhibits completely different characteristics, while the distribution of backward signal and pump laser has a similar pattern to what occurred in the forward pumping configuration. Another obvious difference is that the propagating laser power gradually rises to a higher level at the fiber’s pumped end. Since the input pump power and maximum signal power locate at the same side of the fiber, the pumped end will bear a higher thermal load. In addition, the population density at upper level is more flat than that of the forward one, which means a better gain uniformity. However, the disadvantage is also obvious. As the pumped end is also the output end, hence it requires a dichroic mirror at an angle of 45 degree to separate the laser and pump lights. Undesirably, this additional requirement makes the optical system more complex.

### Bi-direction pumping configuration

**Figure 4:**
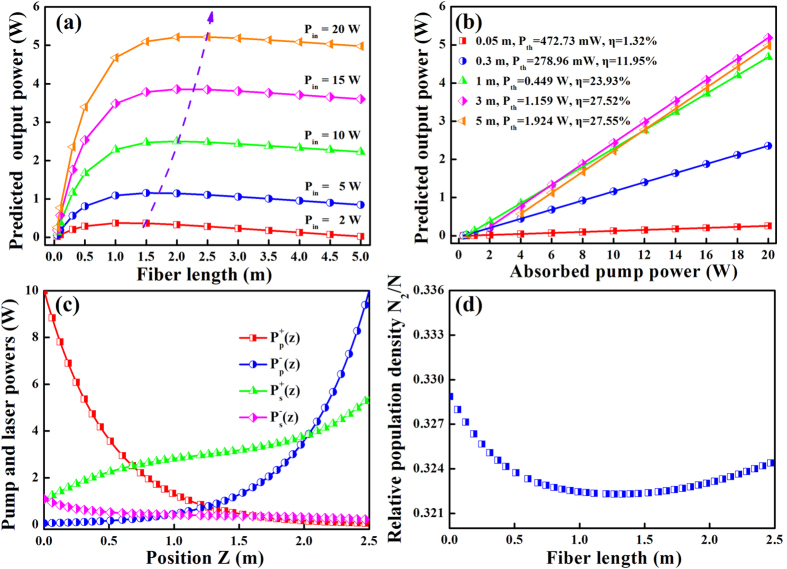
Output power versus (**a**) fiber length for different pump powers, and (**b**) pump power for different fiber lengths. (**c**) The distribution of pump and laser powers, as well as (**d**) the relative population density along the fiber length in bi-direction pumping configuration.

Besides the first two types of pumping configurations, the bi-direction pumping configuration is also frequently used in the design of laser cavity. With the same pump power and fiber length, the laser performance of this EDTF is investigated and the result is shown in Fig. 4. Bi-direction pumping configuration is a combination of forward and backward pumping configurations. Therefore, the maximum slope efficiency of bi-direction pumping configuration is basically between the forward and backward pumping configurations, as shown in Fig. 4(b). It is worth noting that the threshold power reaches 472.73 mW when the fiber is 0.05 m, which is slightly greater than forward (469.70 mW) and backward (469.70 mW) pumping configurations, respectively. In this pumping configuration, the distribution of pump power is more flat than other pumping configurations. Namely, in situations where the fiber is pumped by high-power laser sources, it will carry a lower thermal load than any other pumping configurations. This is very beneficial to maintain a stable operation for fiber lasers with high-power output.

**Figure 5:**
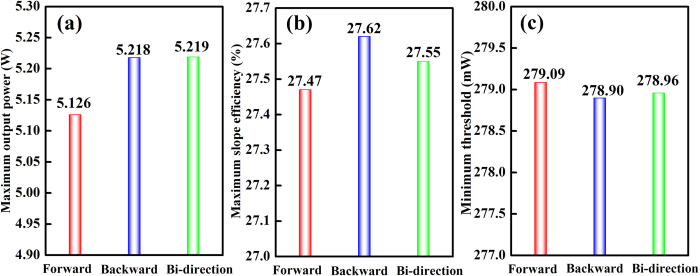
The comparison of predicted (**a**) maximum output power, (**b**) maximum slope efficiency, and (**c**) minimum threshold of Er^3+^-doped tellurite fiber lasers under different pumping configurations. Pump power: 20 W, fiber lengths: 0.05–5 m.

Figure 5 shows the comparison of predicted maximum output power, maximum slope efficiency, and minimum threshold of Er^3+^-doped tellurite fiber lasers under different pumping configurations. When the pump power is 20 W with the fiber lengths of 0.05–5 m, the maximum output power P_out_, maximum slope efficiency η, and minimum threshold P_th_ generally follow the below trends:


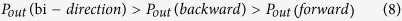



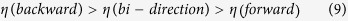



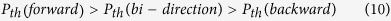


Roughly, the bi-direction pumping configuration can produce a maximum output power under the same conditions, while its slope efficiency and threshold are moderate. Forward pumping configuration does not show any advantages in the aspect of output power and slope efficiency, but it owns a simple optical system. Backward pumping configuration has a smallest threshold and its slope efficiency is larger than that of any other pumping configurations. From what has been discussed above, we may safely draw the conclusion that each potential pumping configurations has its unique advantages and unavoidable shortcomings, which depends largely on the required levels of performance. Besides that, in a practical application, the complexity, stability, and practicality of the laser cavity should also be considered properly when design different types of pumping configurations.

**Figure 6:**
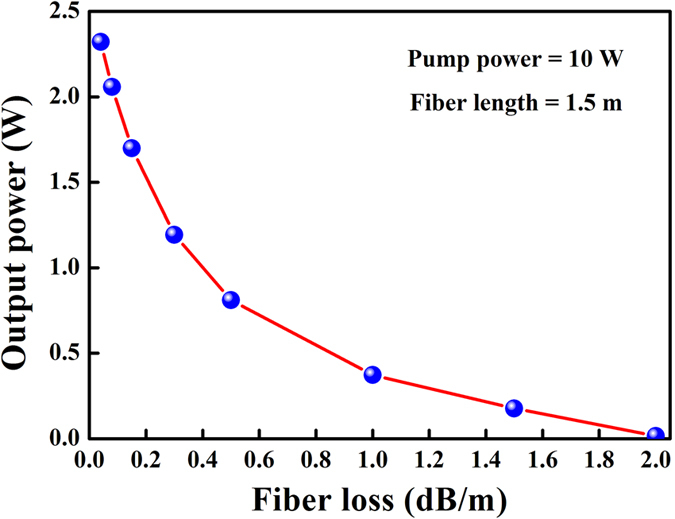
The effect of fiber loss on output power in forward pumping configuration for L = 1.5 m and pump power = 10 W.

Figure 6 shows the effect of fiber loss on output power in forward pumping configuration for L = 1.5 m and pump power = 10 W. As can be seen, the output power sharply decreases from 2.32 W to 16.8 mW with the fiber loss increasing from 0.04 dB/ m to 2 dB/ m. Moreover, it can be found that reasonable output power is expected for fiber loss below 2 dB/ m. According to the literature, the lowest loss for a multimode tellurite fiber currently has decreased to as little as 0.02 dB/ m at 1.56 μm^20^. This means efficient 2.7 μm laser can be achieved in the barium tellurite glass fiber with lower loss by using just the present preparation technology.

### Er^3+^-doped ZBLAN and tellurite fiber lasers

**Figure 7:**
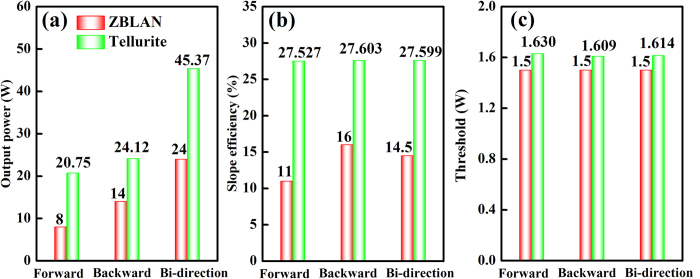
The comparison of (**a**) output power, (**b**) slope efficiency, and (**c**) threshold of Er^3+^-doped ZBLAN^21^ and tellurite fiber lasers under different pumping configurations.

In order to further evaluate the laser performance of this Er^3+^-doped tellurite glass fiber, a preliminary comparison of output power, slope efficiency, and threshold between Er^3+^-doped ZBLAN (performed by Tokita *et al*.)^21^ and tellurite fiber lasers under different pumping configurations are made, as shown in Fig. 7. It should be mentioned that Tokita *et al*. have not optimized the Er^3+^: ZBLAN laser system such as couple mirrors and fiber length, hence some major experiment parameters should kept consistent in order to make a comparison of these two different fiber lasers, for example, by using same dichroic mirrors, fiber lengths, and input powers. The fiber lengths are both set to be 4.2 m, the dichroic mirrors have a high transmittance (95%) at pump wavelength and a high reflectance (99.8%) at lasing wavelength. The maximum pump powers of two directions are 77 and 89 W, respectively. It can be found that tellurite glass fiber may exhibit significant advantages in output power and slope efficiency, about two times greater than ZBLAN fiber, while their threshold powers are very close. Although the eventual conclusion is still need to get the experiment certification further, it can be inferred that this Er^3+^-doped tellurite fiber is an excellent alternative to conventional fluoride fiber for the development of efficient 2.7 μm fiber lasers. This result can be roughly analyzed by comparing the basic properties of ZBLAN^22^ and tellurite glasses, as listed in Table 2. In general, the glass composition leads to big differences in density and refractive index, while the emission cross section of rare earth ions is proportional to the glass refractive index^23^. Because the stimulated emission cross-section is defined as the intensity gain of a laser beam per unit of population inversion when no saturation effects are present or no excited state absorption processes occur, thus higher emission cross section ultimately contribute to obtaining a large possibility of laser output^24^. Although the phonon energy of barium tellurite glass is larger than that of ZBLAN glass, the required phonons for bridging the energy gap between ^4^I_11/2_ and ^4^I_13/2_ levels (3700 cm^−1^) is only slightly less than the latter (5 and 6 phonons, respectively). As the number of phonon required to bridge the gap is similar and big, the non-radiative decay becomes less likely to occur and consequently the radiative transition rate increases^25^. Hence it would be assumed that the small difference of phonon energy between these two glass hosts may play a relatively minor role in this case. On the other hand, the residual hydroxyl (OH^−^) content in the glass has a great effect on lifetime and fluorescence intensity of Er^3+^, which can ultimately leads to the deterioration of laser performance and even inhibits the laser output^26^. Moreover, fiber quality is another essential factor to determine the performance of fiber lasers. Fiber structure, cooling device, and operation technology also have a big impact on the high-efficient laser output.

### Mode field distributions

**Figure 8:**
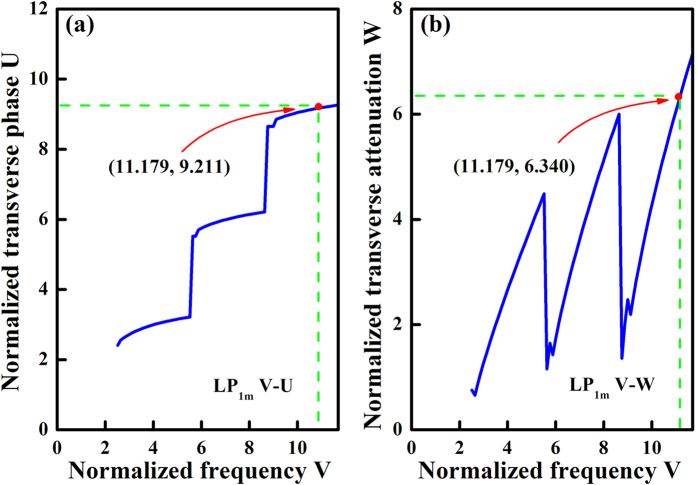
The (**a**) V–U and (**b**) V–W curves of the multimode step-index tellurite fiber.

As we all know, single mode fiber only has one LP_01_ mode, while the multimode fiber possesses more high-order modes. In order to understand the spatial distribution of oscillation modes, the two- and three-dimensional laser field distributions are further analyzed in this multimode step-index tellurite fiber. Firstly, the normalized frequency V, normalized transverse phase parameter U, and normalized transverse damping parameter W are solved by their characteristic equations and Bessel functions^27^. Figure 8 presents the V–U and V–W curves of the multimode tellurite fiber. It is found that the normalized transverse phase parameter U and normalized transverse damping parameter W sharply increases at two broken points (V = 5.482 and 5.696). The three portions of the curves correspond to the LP_10_, LP_11_, and LP_12_ modes, respectively. According to the basic physical parameters of this tellurite glass fiber, the normalized frequency V is calculated to be 11.179, thus the values of U and W can be determined to be 9.211 and 6.340, respectively.

**Figure 9:**
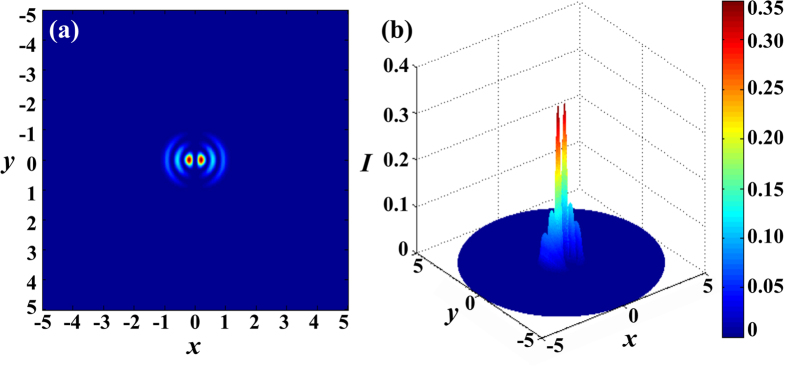
The (**a**) two- and (**b**) three-dimensional laser field distributions of the multimode Er^3+^-doped tellurite glass fiber.

Based on these three parameters, the normalized electric field distribution of LP_12_ mode in fiber core and cladding region can be described visually. Figure 9 presents the two- and three-dimensional laser field distributions of the multimode Er^3+^-doped tellurite glass fiber. As can be seen, the LP_12_ mode has six nearly degenerate modes (two main lobes and four side lobes) in the glass fiber with perfect circular symmetry. This degeneracy of the orthogonally polarized modes easily leads to the mode coupling among them, inducing depolarization after a short propagation length assuming a linearly polarized input. In a polarization maintaining fiber, the degeneracy can be broken by introducing a noncircular symmetry and leads to much improved polarization preservation^28^. Furthermore, it should be noted that the intensity of side lobes is less than one-third of main lobes, which indicates that the energy is mainly centered in the fiber core region, as shown in Fig. 9(b).

When considering the multimode fiber operation in the modeling and in the mode competition, some technical parameters such as mode separation, diffraction limit, and absorption boundary should be added into the equations. Mode separation represents the difference of frequencies between two successive longitudinal modes in both core and cladding area in the multimode fibers. The diffraction algorithm limit involves the degree of diffraction, which occurs for pump and laser waves inside the core area. The absorption boundary shows the limited size of pumping beam, in which the pump spot size is larger than the core diameter. In addition, another limitation of this model is that fully comprehensive commands are not yet included to express other possible luminescence mechanisms of fiber lasers and related thermal effects. All of these will make the simulation program more complex and work for long hours^29^, so we only perform a preliminary simulation to describe the spatial distribution of oscillation modes in this multimode Er^3+^-doped tellurite fiber. On the other hand, our ultimate aim is to realize the 2.7 μm laser from a single mode tellurite fiber for obtaining excellent laser beam quality and slope efficiency, which can be considered from the aspects of redesign core size and numerical aperture of the fiber.

## Conclusions

In summary, the feasibility of an Er^3+^-doped tellurite fiber lasers operating at 2.7 μm has been theoretically predicted. Based on the rate equations and propagation equations, the effects of pumping configuration and fiber length on laser output power, slope efficiency, threshold, and intracavity pump and laser power distributions have been analyzed in detail. When the pump power is 20 W, a maximum output power of 5.219 W from a 2.5 m long fiber is obtained in a bi-direction pumping configuration. Maximum slope efficiency reaches 27.62% in a backward pumping configuration with a 5 m long fiber. The forward pumping configuration allows a threshold of 469.70 mW using a 0.05 m short-length fiber. It is also found that reasonable output power is expected for fiber loss below 2 dB/m. Furthermore, it is found that six nearly degenerate modes (two main lobes and four side lobes) exist with perfect circular symmetry by simulating the two- and three-dimensional laser power distributions in the core and cladding region. Our results indicate that the Er^3+^-doped barium tellurite fiber is a promising candidate for efficient mid-infrared fiber lasers and exhibits a significant advantageous performance over the classical fluoride fiber.

## Additional Information

**How to cite this article**: Wang, W. *et al*. Numerical analysis of 2.7 µm lasing in Er^3+^-doped tellurite fiber lasers. *Sci. Rep.* **6**, 31761; doi: 10.1038/srep31761 (2016).

## Figures and Tables

**Table 1:** Basic physical parameters of Er^3+^-doped tellurite glass fiber lasers.

Symbol	Value	Unit
λ_p_	808	nm
λ_s_	2.7	μm
τ	3.09	ms
σ_ap_ = σ_ep_	1.69	10^−21^ cm^2^
σ_as_	3.76	10^−21^ cm^2^
σ_es_	7.93	10^−21^ cm^2^
A_c_	5	10^−5^ cm^2^
N	3.56	10^20^ cm^−3^
α_p_	1.38	10^−3^ cm^−1^
α_s_	1.08	10^−5^ cm^−1^
L	0.05–5	m
Γ_p_	0.83	—
Γ_s_	0.9	—
R_1_	0.99	—
R_2_	0.04	—

**Table 2:** The comparison of basic properties of Er^3+^-doped ZBLAN and tellurite glass.

Glass	**ρ** (g·cm^−3^)	n	ħω (cm^−1^)	A_rad_	**β** (%)	**τ** (ms)	**σ**_**es**_ (10^−20^ cm^2^)	J-O parameters (10^–20^ cm^2^)		
								Ω_2_	Ω_4_	Ω_6_
ZBLAN^22^	4.38	1.499	586	28.8	15.85	5.5	0.66	3.27	1.3	1.75
Tellurite	5.43	2.045	770	50.84	16	3.09	0.79	6.21	1.9	0.06
